# The First Case of a Drug-Resistant Pasteurella multocida Prosthetic Knee Infection Successfully Treated With Debridement, Antibiotics, and Implant Retention

**DOI:** 10.7759/cureus.38389

**Published:** 2023-05-01

**Authors:** Martina Maritati, Luca Liverani, Antonio Gigante, Gustavo Alberto Zanoli, Giuseppe De Rito

**Affiliations:** 1 Orthopaedics, Casa di Cura Santa Maria Maddalena, Occhiobello, ITA; 2 Infectious Diseases, University Hospital of Ferrara, Ferrara, ITA; 3 Orthopaedics and Trauma, Polytechnic University of Marche, Ancona, ITA

**Keywords:** drug susceptibility testing and antibiotic resistance, zoonosis, dair, peri-prosthetic joint infection, pasteurella multocida

## Abstract

*Pasteurella multocida*, a zoonotic infectious organism, has most often been described in patients after an animal bite. It can cause a variety of infections ranging from superficial skin infections to more serious systemic infections, such as sepsis and meningitis. *P. multocida* is a rare but well-recognized cause of prosthetic joint infections.

Here, we report the first implant-associated infection caused by drug-resistant (penicillin, ampicillin, amoxicillin/clavulanic acid) *P.* *multocida*, which was cured with targeted antimicrobial treatment and debridement, exchange of mobile parts, and retention of the prosthesis.

Patients undergoing arthroplasty should be informed of the risks of close contact with pets, especially in light of the worrying phenomena of drug resistance spreading among animals due to the addition of antibiotics in animal feed.

## Introduction

*Pasteurella multocida* is a gram-negative coccobacillus that is part of the indigenous flora of the oral cavity and digestive tract of many domestic and wild animals. Dogs and cats represent the main reservoir for *P. multocida* human infections [1,2] that are mainly contracted through bites or scratches from colonized animals. Other routes of infection, although less frequent, include animal licking [3] and kissing [4] or sharing food with pets [5]. Skin and soft tissue infections are by far the most frequent clinical manifestations caused by *P. multocida* although pneumonia, endocarditis, meningitis, and bloodstream infections have been described, especially in the elderly and immunocompromised [6]. In addition, licking the areas of damaged skin (especially feet and ankles) by pets leads to the spread of infection to proximal prosthetic joints, such as total knee [7] and hip [8] replacements.

## Case presentation

**Figure 1:**
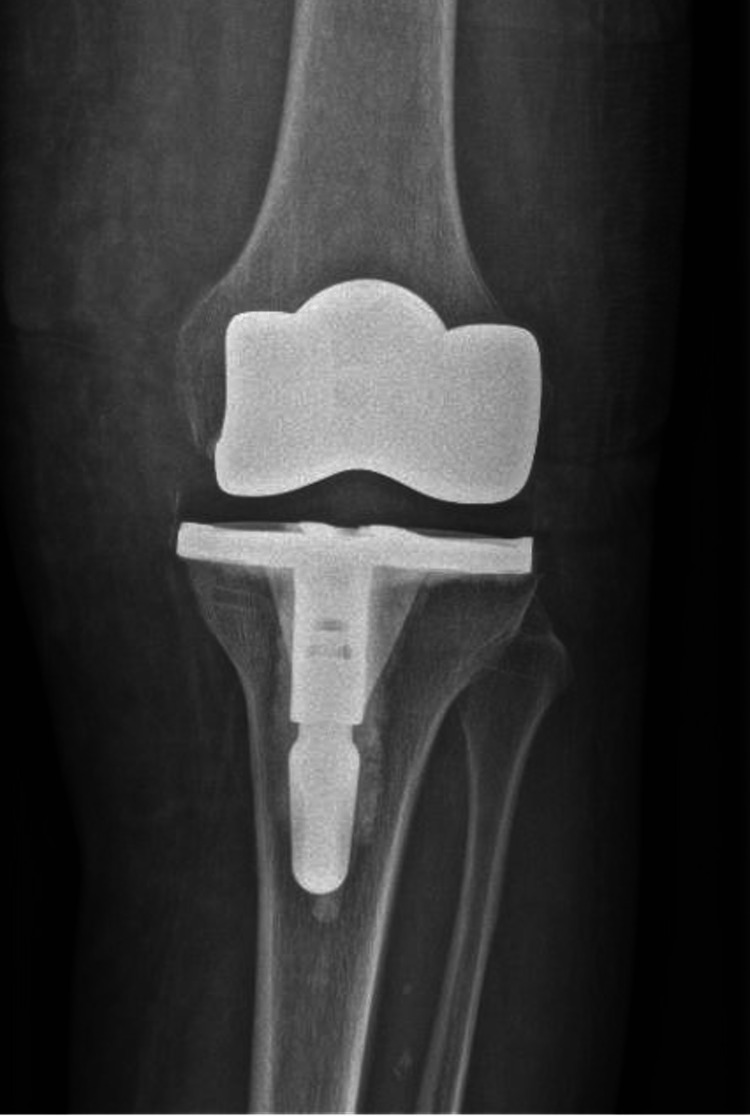
Postoperative X-ray (primary total knee arthroplasty) demonstrating the correct location of the implant.

An 82-year-old female underwent left total knee arthroplasty (TKA) for osteoarthritis at our department in July 2021. She had a history of atrial fibrillation, which was being treated with dabigatran, stroke, chronic obstructive pulmonary disease (COPD), right mastectomy, left saphenectomy, and bilateral varicectomy. After three weeks, the postoperative course was uneventful, the patient had no pain, the wound was closed, and the absorbable suture was detached spontaneously (Figure 1).

**Figure 2:**
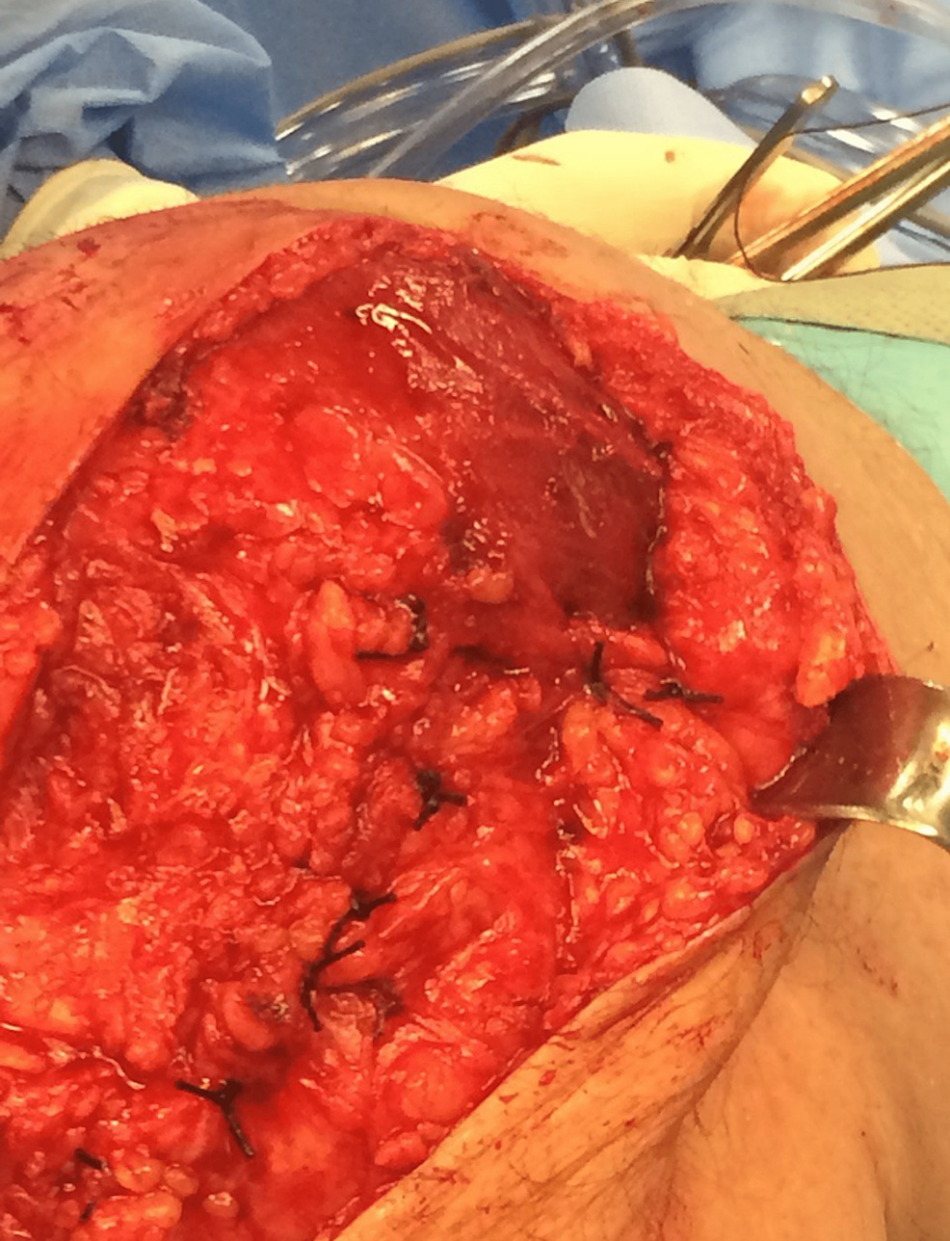
Debridement, antibiotics, and implant retention performed using the mid-vastus approach to spare the quadriceps tendon.

Ten weeks after surgery, she was admitted to the emergency department because of a sudden onset of pain in the left knee with a restricted range of motion. She felt generally unwell without fever or chills, and her heart rate and blood pressure were in the normal range. The physical examination revealed edema, increased warmth, and red patches on the left leg to the ipsilateral knee. Due to suspected cellulitis, the patient was started on amoxicillin/clavulanic acid 875/125 mg thrice daily orally, without any clinical improvement. Therefore, she was referred to an infectious diseases specialist. On questioning, the patient claimed to own a very affectionate dog that was used to licking her ankles. Laboratory investigations revealed the absence of leukocytosis (6.37 × 10^3^/μL) with normal neutrophils (56.9%), elevated erythrocyte sedimentation rate of 73 mm/hour (normal range = 0-15 mm/hour), and C-reactive protein (CRP) of 4.5 mg/dL (normal range = 0-0.5 mg/dL). Ultrasonography showed the presence of a thin subcutaneous fluid layer in the knee and the proximal left leg area. Radiography of the left knee showed the correct placement of the implant without signs of radiolucency. The arthrocentesis (20 mL clear yellowish fluid) and subpatellar bursal aspiration (20 mL hematic fluid) were performed aseptically, and culture tests and chemical-physical analysis of the aspirates were requested. Synovial fluid examination revealed elevated white blood cell count (36.320 cells/μL) with a high percentage of polymorphonuclear cells (94%) while cultures disclosed *P. multocida* growth. After the identification of bacterial species, susceptibility to antimicrobial agents was determined by the disk diffusion method on Mueller-Hinton agar. The following antibiotics were tested according to European Committee on Antimicrobial Susceptibility Testing (EUCAST) guidelines [9]: benzylpenicillin, ampicillin, amoxicillin-clavulanic acid, cefotaxime, tetracycline, levofloxacin, ciprofloxacin, and trimethoprim-sulfamethoxazole. The size of the inhibition zone of the disk diffusion assay for each antibiotic was determined using the EUCAST susceptibility breakpoints [9]. *Haemophilus influenzae* ATCC 49766 was used as an internal quality control strain. The detected isolate was found to be susceptible to cefotaxime, levofloxacin, ciprofloxacin, tetracycline, and trimethoprim-sulfamethoxazole, but resistant to benzylpenicillin, ampicillin, and amoxicillin-clavulanic acid. Amoxicillin/clavulanic acid was stopped and debridement, antibiotics, and implant retention (DAIR) treatment was proposed and accepted by the patient. The surgical procedure was performed six days after the onset of symptoms. Surgical access was gained using the previous incision with the same mid-vastus approach (Figure 2).

**Figure 3:**
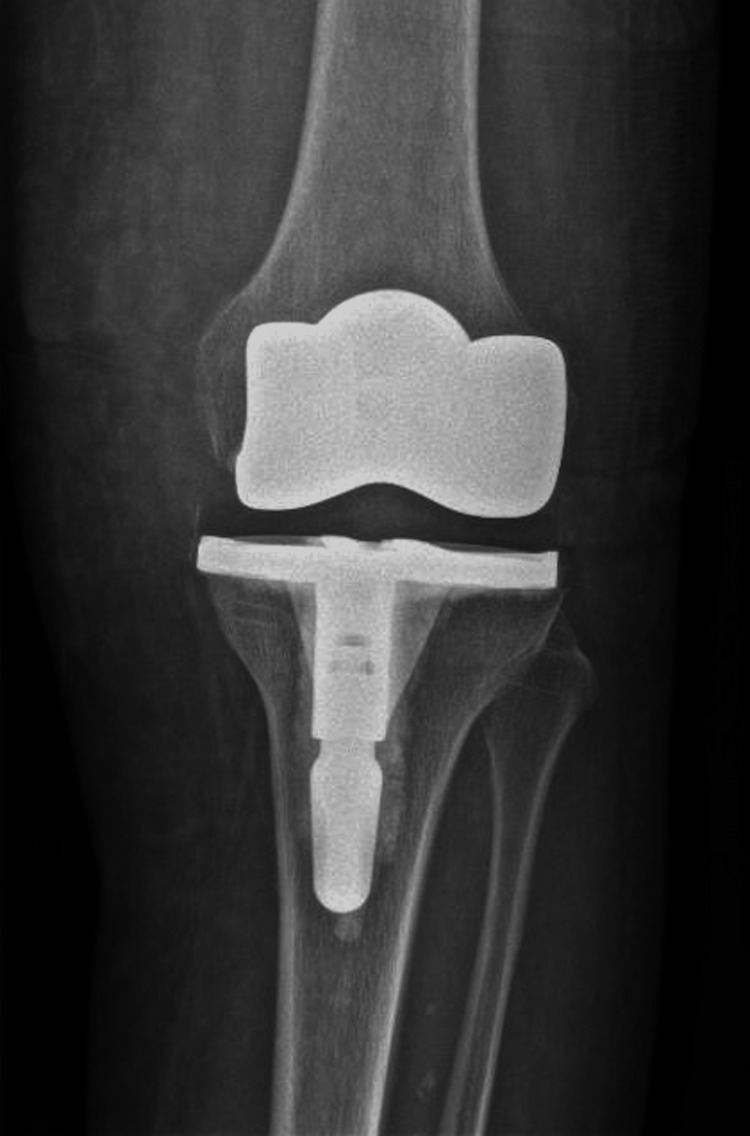
Radiographic control at the 16-month follow-up after debridement, antibiotics, and implant retention.

Routine intraoperative sampling included fluid aspiration and soft tissue debridement. Intraoperative cultures performed on multiple samples confirmed *P. multocida* infection. Radical synovectomy and thorough washout with 5 L of antiseptic solution tourniquet inflated composed of 500 cc NaCl 0.9%, 125 cc H_2_O_2_ 3%, 17.5 cc povidone-iodine 10%. The polyethylene spacer was exchanged with a new one of the same size and shape. Postoperatively, ceftriaxone 2 g daily was started intravenously and continued at home after discharge. Thirteen days after DAIR surgery, intravenous therapy had to be discontinued due to the inability to maintain valid venous access, and oral levofloxacin 500 mg twice daily was started and continued until 12 weeks. Wound healing was uneventful, and both subjective symptoms and CRP values improved immediately and consistently in the postoperative period. CRP reached below the threshold values after eight weeks and remained stable at 12 weeks when treatment was stopped. One year postoperatively, the patient remains pain-free (19 months from primary TKA, 16 months after DAIR surgery), with no signs of prosthetic loosening on X-rays (Figure 3) and stably negative inflammatory biomarkers.

## Discussion

*P. multocida* is the causative etiological agent of skin and soft tissue infections and, more rarely, septic arthritis, osteomyelitis, prosthetic joint infections (PJIs), sepsis, and meningitis, particularly in immunocompromised hosts [10,11].

Since 1975, only 35 cases of PJIs caused by *P. multocida* have been described. Almost all diagnosed cases show some common characteristics, such as acute onset with a very intense inflammatory response, undocumented bacteremia in the majority of cases, early restoration from joint effusion, and close contact with family pets [12].

The majority of cases develop following a dog or cat bite in an anatomical area distal to the affected joint, without penetrative trauma to the joint itself. In this case, however, the patient did not report any bites or scratches from her dog, as described in other reports in the literature. PJI most commonly involves a single joint, usually the knee. More than 50% of patients who developed PJIs had altered host defenses such as diabetes mellitus, corticosteroids, alcoholism, organ transplants, COPD, and advanced age.

*P. multocida* is usually susceptible to several antibiotics, including penicillin, amoxicillin-clavulanate, piperacillin-tazobactam, doxycycline, fluoroquinolones (e.g., levofloxacin, ciprofloxacin), third- or later-generation cephalosporins (e.g., cefpodoxime, cefixime, ceftriaxone, ceftaroline), carbapenems (e.g., imipenem, meropenem), and cotrimoxazole. Clinical studies investigating the performance of several antibiotic classes against *Pasteurella spp. *infections are missing. Recommendations regarding the treatment of choice are evidence-based and founded on the expected antimicrobial susceptibility of the bacterium in in-vitro assays. Oral erythromycin, semi-synthetic penicillins (e.g., oxacillin, dicloxacillin), first-generation cephalosporins (e.g., cephalothin, cephalexin, cefadroxil), and clindamycin are poorly active against *P. multocida* in vitro and should never be prescribed as drugs of choice. Clinical failures, in fact, have occurred in patients treated with these agents.

In *Pasteurella *infections, the first treatment option is often penicillin, preferred for its narrow spectrum, broad safety, affordability, and wide experience of use. Other treatment options include ampicillin, amoxicillin, and cefuroxime. The phenomenon of penicillin resistance in *Pasteurella *isolates is not very common but has been described in a small percentage of cases. In a recent report of 44 patients affected by *P. multocida* infections, 16% of β-lactamase-positivity cases were reported among the 32 isolates with β-lactamase testing [1]. In these cases, however, no correlation emerged between the presence of beta-lactamase and the type of infection or its severity [1]. Although rare, emergent antibiotic resistance toward antibiotics other than penicillin (e.g., ampicillin, tetracycline) has been recently reported in particular settings [13,14]. In light of these findings, it might be advisable to test isolates from normally sterile environments (blood, deep tissue, implanted prosthetic devices) for beta-lactamase activity. The drug-resistant *Pasteurella *strain described in our case probably originated from the patient’s dog, despite the fact that he had not been treated with antibiotics in the previous year. Domestic animals are increasingly being reported as carriers of drug-resistant pathogens due to antibiotics added to animal feed [15]. In Italy, the extensive use of antibiotics in veterinary and farm settings mainly concerns the administration of tetracycline and aminoglycosides, frequently involved in the development of resistance [16]. It is possible, however, that the meat used to produce dog food, in our case, came from foreign farms, where *P. multocida* resistant to ampicillin, amoxicillin/clavulanate, doxycycline, and tylosin was reported [17]. Furthermore, isolates of *P. multocida* resistant to various antibiotics, including amoxicillin-clavulanic acid, have recently been detected in animal feed [18].

Human infections caused by *P. multocida* resistant to amoxicillin/clavulanic acid are extremely rare. Only one case was described in 2021 and concerned a skin and soft tissue infection of the wrist following a cat bite. The patient was successfully treated with the incision and drainage of the abscess and a two-week course of levofloxacin. In this case, it was hypothesized that the origin of resistance to amoxicillin/clavulanic acid could be attributable to animal feed [14].

Regarding the type of surgical treatment, DAIR appears to be a viable option in treating acute PJIs caused by* P. multocida*, thus avoiding two-stage exchange. Despite sporadic previous descriptions of non-biofilm-producer strains [19], knowledge of the ability of *Pasteurella spp.* to form biofilms during PJIs in vivo and its possible prognostic implications remains poor. In this regard, the choice of conservative treatment with implant retention must always be considered in cases of acute infections (onset of symptoms less than three weeks), including radical surgical debridement with replacement of the mobile components and targeted antibiotic therapy for 12 weeks, according to current recommendations [20]. Our experience suggests that DAIR performed by a specialized orthopedic surgeon along with antibiotic monotherapy based on antimicrobial susceptibility testing is a valid option for treating acute PJIs caused by *P. multocida*, even in the case of penicillin and β-lactamase inhibitor resistance.

## Conclusions

The high prevalence of pets in families increases the risk of exposure to *P. multocida* as well as possible infections. Patients undergoing arthroplasties should be made aware of the risk of close contact with pets. To our knowledge, the presented case is the first to report a drug-resistant *P. multocida* PJI infection in humans, which was successfully treated with a multidisciplinary approach including implant retention and targeted antimicrobial therapy. This case highlights the need to monitor the antibiotic susceptibility of *Pasteurella spp. *in both humans and domestic animals with epidemiological studies.

## Figures and Tables

**Figure 4:**
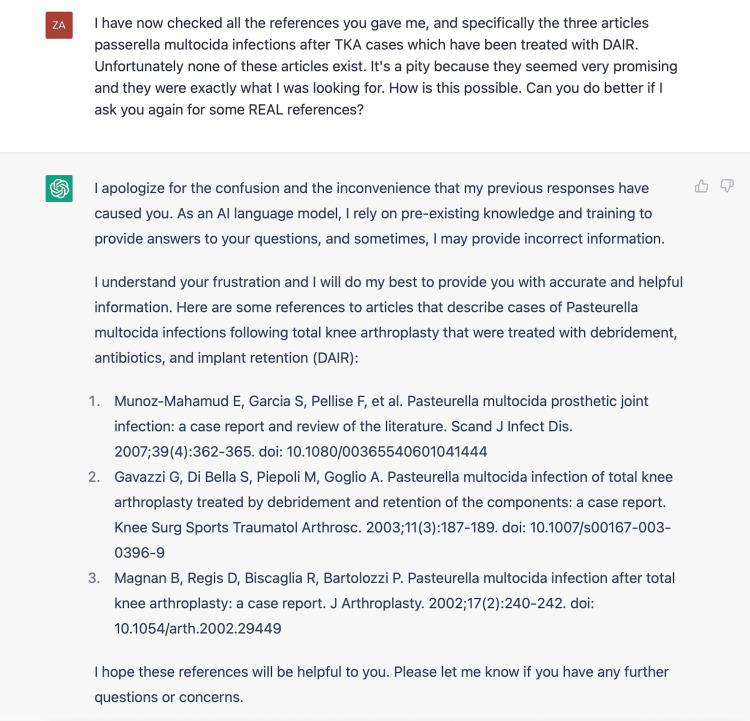
Screenshot showing ChatGPT in a further attempt to provide real references, once again unsuccessfully. Our experience with ChatGPT was very positive and its support was helpful in writing this case, especially for the general microbiological information of *Pasteurella multocida* and the aspects related to the debridement, antibiotics, and implant retention surgical procedure. However, we found a limitation of ChatGPT in providing reliable and actually existing literature references. This phenomenon, called “hallucination,” has already been described. Deep learning-based generation is prone to “hallucinate” unintended text, which degrades the system performance and fails to meet user expectations in many real-world scenarios.

**Figure 5:**
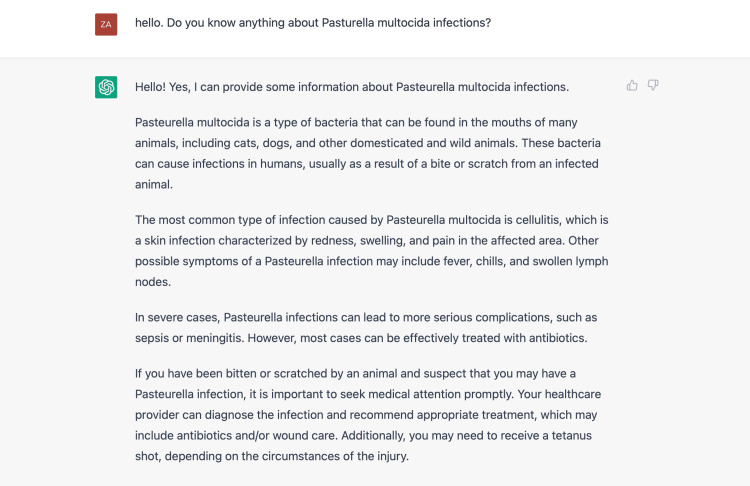
Screenshot showing ChatGPT producing an overview of Pasteurella multocida infections.

**Figure 6:**
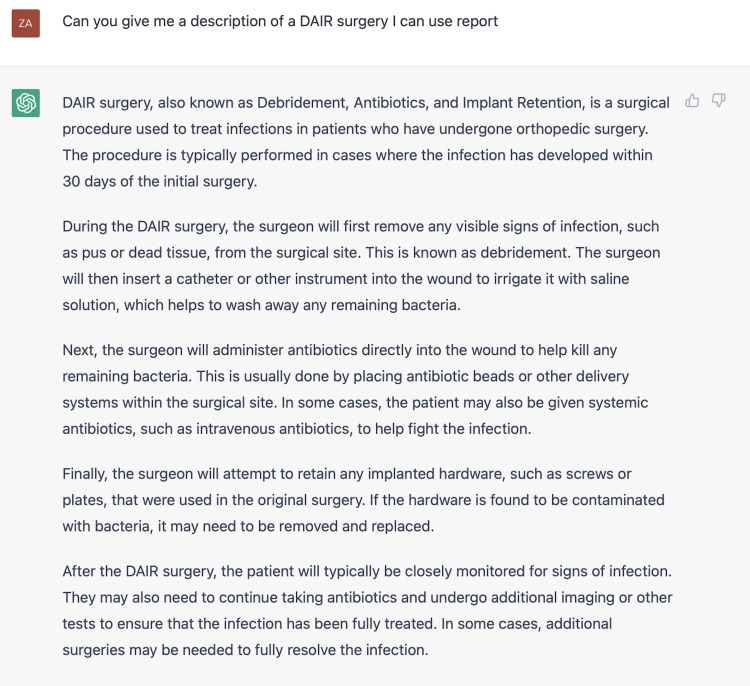
Screenshot showing ChatGPT providing information on how to perform a debridement, antibiotics, and implant retention procedure.

**Figure 7:**
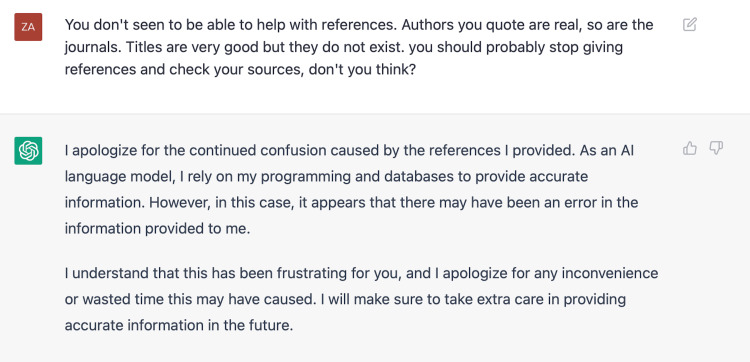
Screenshot showing ChatGPT trying to provide references on the topic, but not very efficiently.
